# Temporal perception is distorted by submaximal and maximal isometric contractions of the knee extensors in young healthy males and females

**DOI:** 10.3389/fspor.2023.1185480

**Published:** 2023-07-26

**Authors:** Hayley R. Gardner, Andreas Konrad, Shahab Alizadeh, Andrew Graham, David G. Behm

**Affiliations:** ^1^ School of Human Kinetics and Recreation, Memorial University of Newfoundland, St. John’s, NL, Canada; ^2^ Institute of Human Movement Science, Sport and Health, Graz University, Graz, Austria; ^3^ Department of Kinesiology, University of Calgary, Calgary, AB, Canada

**Keywords:** time processing, arousal, verbal time estimation, intensity, exercise

## Abstract

**Introduction:**

The estimate of time (temporal perception) is important for activities of daily living, sports and even survival, however time perception research needs greater scrutiny. Time estimation can influence movement decisions and determine whether the individual is successful at their goal, The objectives of this study were to examine participants perception of time at 5-, 10-, 20-, and 30-s intervals to determine possible distortions of time estimates caused by varying intensity isometric contractions, and sex differences.

**Methods:**

In this repeated measures study, 19 participants (10 females, 9 males) endured two sessions, which consisted of a cognitive task of estimating time intervals while performing an isometric knee extension at maximal, submaximal (60%), and distraction (10%) intensities and a non-active control. In addition to time estimates; heart rate (HR), tympanic temperatures and electromyography during the intervention contractions were monitored. Maximal contractions induced significantly greater time underestimations at 5-s (4.43 ± 0.93, *p* = 0.004), 20-s (18.59 ± 2.61-s, *p* = 0.03), and 30-s (27.41 ± 4.07-s, *p* = 0.004) than control. Submaximal contractions contributed to time underestimation at 30-s (27.38 ± 3.17-s, *p* = 0.001). Females demonstrated a greater underestimation of 5-s during the interventions than males (*p* = 0.02) with 60% submaximal (−0.64-s ± 0.26) and distraction (−0.53-s ± 0.22) conditions. For the other 10-, 20-, 30-s intervals, there was no significant time perception sex differences. The control condition exhibited lower HR (75.3 *± *11.6) than the maximal (92.5 *± *13.9), 60% submaximal (92.2 *± *14.4) or distraction (90.5 *± *14.7) conditions. Tympanic temperatures were not influenced by the contraction intensities.

**Discussion:**

There was greater integrated knee extensor electromyographic activity during the maximal contractions to suggest greater neuromuscular activation that may influence time perception. However, there was no consistent effect of changes in HR or temperature on time estimates. This work adds to the growing literature of time perception during exercise to state that time is significantly underestimated when performing moderate to vigorous intensity exercise.

## Introduction

Temporal perceptions are critical for everyday behaviour and survival (1), yet the perception of time is underexplored. Time estimation can influence movement decisions and determine whether the individual is successful at their goal, for example, the 25-s time restriction for a tennis serve, pacing in endurance races, or time line violations in basketball. Time perception is a subjective individual judgment of a pre-determined chronological interval (2). Evaluating one's perception of time can be achieved by comparing chronological time (objective time) to one's perception (subjective time). The Scalar Expectancy Theory (SET) (3) and the Striatal Beat Frequency (SBF) (4) model attempt to explain the human experience of time perception. The SET proposes that a theoretical pacemaker produces a series of pulses, and an accumulator counts the number of pulses emitted over time. The monitoring of pulses determines the duration experienced. This is known as a clock phase, followed by a memory stage where the value of pulses in the accumulator is compared to previously stored durations leading to a temporal decision and response (3). The neurobiology of the SBF model uses alterations to cortico-striatal networks to influence the theoretical internal clock to cause temporal distortion (4). However, both the SBF and SET are influenced by arousal, which can result in temporal distortions (physiological or psychological) (5–8).

The psychological factor of attentional effect plays a critical role in time perception when encountering a dual-task; the ability to perform two tasks simultaneously. Dual-tasking measures a component of executive function as participants must coordinate their attention to both tasks performed (9). Hanson and Lee (10) found a mismatch in performance during a dual-task, such that the cognitive task was given less attention than the physical task. The conclusion was that the perception of time increased while the physical performance of the task decreased. Presently, studies have found conflicting evidence for temporal distortions as it seems to depend on the distraction. For example, Lontz (11) found that an auditory distraction (10-s of an intermittent Morse code sound at 60 decibels) led to an overestimation of retrospective and prospective time. In contrast, the distraction of music has shown that participants can perform tasks for longer durations (12, 13). To our knowledge, limited studies have investigated time perception during a distraction; therefore, future research is needed in this area.

Exercise has been shown to increase physiological arousal, such as increased heart rate, temperature, and neuromuscular responses such as increased electromyographic (EMG) activity (representative of motor unit recruitment and rate coding) (14, 15) as well as psychological arousal such as increases in perceived exertion, competitive tension, and other factors (16). Although, the influence of exercise on temporal perception is currently limited. However, researchers found high-intensity scenarios, similar to exercise arousal levels, such as front-line healthcare workers overestimated time to aid a cardiac arrest case (17). This accuracy of temporal events often seen in sports may be due to the practice and thus knowledge of the task. Tobin and Grondin (18) instructed elite swimmers to complete their strongest and weakest strokes while estimating the time to complete each stroke. The swimmers were more precise at estimating time completion with their strongest stroke and overestimated the weaker stroke. One's perception of time may be easily influenced by the environment and related to their awareness of the task's psychological and physiological factors (19).

The difference in contraction types (i.e., dynamic and isometric) and intensities has been underreported in time perception research. A single study concluded that performing an isometric grip test can distort time intervals (20). Hanson and Lee (21) reported a higher rating of perceived exertion score (RPE: indicator of perceived exercise intensity) resulted in a compressed perception of time during a 30-min treadmill protocol. The type and intensity of exercise and perception of time research is limited.

Additionally, exercise can interact with many factors such as age, sex, and fitness level. Presently, only one study conducted by Hanson and Buckworth (22) attempted to investigate sex differences and temporal distortion during self-paced running. The study found that females and males differed during a self-paced exercise; females underestimated time while males overestimated the temporal intervals. These factors can help understand the physiological and psychological influence of time perception that will contribute to human experience and performance.

The goal of this study was to analyze time perception in intervals of 5-, 10-, 20-, and 30-s when performing isometric knee extensions at varying intensities (maximal, 60% of maximal, and 10% of maximal). Furthermore, this study also aimed to explore sex differences in time perception. Based on prior literature (23), it was hypothesized that time perception (estimates) would be impaired to a greater degree with longer chronological time. Additionally, it was hypothesized that maximal and submaximal exercise intensities may distort temporal perception to a greater extent due to increased arousal than the control and distraction conditions with the attentional demands required (4–8). Lastly, we hypothesized that females would tend to distort time intervals to a greater extent than male participants (23).

## Materials and methods

### Participants

Based on an “a priori” statistical analysis (G*power version 3.1.9.2, Dusseldorf Germany) and a pilot project (11 participants), it was determined that approximately 15 participants were needed to achieve an alpha of 0.05 and a power of 0.8. A convenience sample of 19 (9 males, 10 females) healthy physically active participants between the ages 18–30 years were recruited (Table 1). The participants had no history of lower limb injuries in the last 6 months and they resistance trained more than twice a week for over two years. All participants were kinesiology or physical education undergraduate or graduate students who had experience with performing maximal isometric contractions with knee extensions and handgrip dynamometer. Prior to completion of the study, participants read, signed an informed consent document, and completed the Physical Activity Readiness Questionnaire-Plus (Canadian Society for Exercise Physiology, 2020). A COVID-19 pre-screening test was submitted before entry into the laboratory. The study was approved by the institution's Interdisciplinary Committee on Ethics in Human Research (20210782-HR) in accord with the Declaration of Helsinki.

**Table 1:** Anthropometric data of male and female participants, listed as means ± standard deviation.

	Age (years)	Height (m)	Mass (kg)	BMI (kg/m^2^)
Males (9)	23 ± 2.8	1.80 ± 6.3	76.5 ± 8.4	23.7 ± 2.3
Females (10)	23 ± 1.9	1.64 ± 6.9	67.0 ± 14.7	25.1 ± 5.8
Total (19)	23 ± 4.4	1.71 ± 10.3	72 ± 12.7	24.4 ± 4.3

### Experimental design

**Figure 1:**
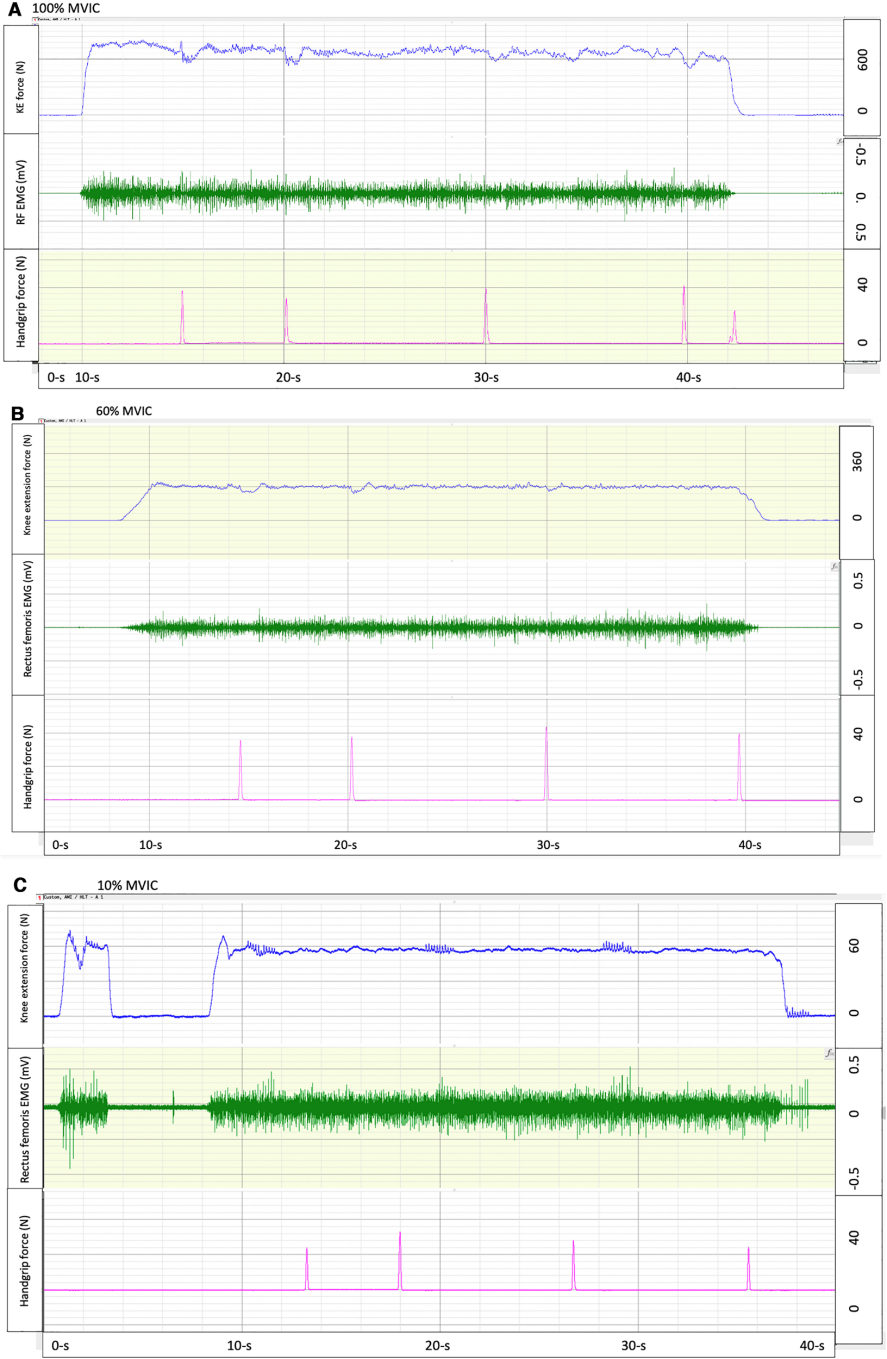
Exemplary force tracings of knee extension isometric contractions at 100% (**A**), 60% (**B**), and 10% (**C**) maximal voluntary isometric contraction (MVIC) force (top row), with electromyography (middle row) and time estimates (5-, 10-, 20- and 30-s) indicated by squeezing a handgrip dynamometer (bottom row).

This repeated measures study design was used to examine the effect of time perception when performing isometric contractions of the knee extensors at varying intensities, as well as to investigate possible sex differences in the perception of time. Participants attended two sessions on two different occasions, separated by at least 48 h, to minimise the carryover effects of fatigue, based on the American College of Sports and Medicine (ACSM) recommendations for exercise recovery (24). The two sessions were randomized (generated by Microsoft Excel) as Session: “Control + 100% MVIC” [control, and 30 s maximal voluntary isometric contraction (MVIC) protocols] or Session “10 + 60% MVIC” (distraction using 10% MVIC and a submaximal contraction at 60% MVIC protocols). Each session included an intervention that involved concurrent activities of a knee extension voluntary isometric contraction (10%, 60% or 100% MVIC) or non-active control while the participant estimated the occurrence of time intervals of 5-, 10-, 20- and 30-s (Figure 1). Heart rate and tympanic temperature were also recorded pre- and post-intervention (before the concurrent contractions and time estimate) and during the intervention protocol. For the first session, anthropometric data were recorded for each participant.

### Session preparation

During the familiarization protocol, participants were seated while they observed the passage of 30-s on a computerized digital clock twice. Then, participants were asked to recall chronological temporal intervals of 5-, 10-, 20-, and 30-s without viewing the digital clock, for six repetitions. The estimate of the time intervals (5-, 10-, 20-, and 30-s) was identified when the participant squeezed a hand dynamometer. A strain gauge (Omega Engineering Inc., LCCA 250, Don Mills, Ontario) embedded in the hand dynamometer was connected to the computer software acquisition system (BioPac AcqKnowledge). When squeezed, force was registered to document the participants' time estimates. The first observable deviation from baseline (sensitivity measured in grams) was used to identify the time estimate. An average of the participant's six time estimate attempts was determined to compare to chronological time during the familiarization and pre-intervention testing. Post-intervention testing involved one estimate of the time intervals during the contraction (10%, 60% or MVIC) or control period.

Time familiarization was followed by EMG electrodes preparation and a warm-up on a cycle ergometer (Monark Inc., Sweden) for five minutes at a cadence of 70 rpm at one kilopond (70 Watts). Participants were then seated in a leg extension machine via a custom-built apparatus (Technical Services Memorial University of Newfoundland), with the knee fixed at 110°, to perform four warm-up isometric knee extensions. A strap was placed around the waist to limit the contribution of upper body movement to the knee extension. Participants were instructed to cross their hands across their chest while holding a hand dynamometer. The dominant ankle, determined by which foot the participant would kick a ball (25), was inserted into a padded ankle cuff attached to a strain gauge (Omega Engineering Inc., LCCA 250, Don Mills, Ontario). Differential voltage (±0.03% linearity and 3 mv/V) from the knee extension and handgrip dynamometer strain gauges, sampled at a rate of 2,000-Hz, were calibrated (to Newtons), amplified (×1,000), digitally converted (Biopac Systems Inc. DA 100 and analog to digital converter MP100WSW; Holliston, MA), and monitored on a computer. A commercial software program (AcqKnowledge III, Biopac Systems Inc., Holliston, MA) was used to analyze the digitally converted analog data.

Participants performed two knee extension MVICs for four seconds with one-minute rest. If the difference between the two MVIC force outputs (Newtons) was more than 5%, a third contraction was performed to ensure the participant's maximal force was achieved. Prior to performing each MVIC, the participant was told to contract their thigh (quadriceps) as hard and fast as possible. The peak force from the 4-s MVIC was used to calculate the intervention submaximal contraction forces (10% and 60% MVIC). The order of the two testing sessions was randomized.

### Session: control + 100% MVIC

For the control protocol, participants sat in a custom-built apparatus (Technical Services Memorial University of Newfoundland), but did not perform concurrent knee extension contractions as they as they estimated 5-, 10-, 20-, and 30-s. Time estimates were marked by the computer software by squeezing the hand dynamometer for each interval. The handgrip contraction intensity was just sufficient to provide a deviation from baseline and thus was of low intensity. Simultaneously, participants announced their RPE (BORG scale) after each time estimate. The MVIC protocol followed the same procedure except the participant contracted as fast and hard as possible for approximately 30-s. During this period, they also squeezed the handgrip dynamometer periodically to demonstrate their estimates of their perceived 5-, 10-, 20-, and 30-s and stated their RPE after every estimation. The control protocol was always conducted first so there would be no fatigue effects on the MVIC condition, whereas the MVIC condition could have adversely affected the control condition. A three-minute rest interval was allocated between the control and MVIC conditions.

### Session: 10 + 60% MVIC

With the distraction and submaximal protocols, 10% and 60% of the participant's MVIC was determined respectively, and a range band of the relative MVIC force (+/−10%) was shown to the participant on the computer screen (AcqKnowledge III, Biopac Systems Inc., Holliston, MA) to gauge the intensity throughout the protocol. Before counting, the participant performed a knee extension to achieve approximately 10% or 60% of their MVIC. Once this was sustainable, the participant started to estimate the time intervals by pressing the hand dynamometer at the 5-,10-, 20-, and 30-s intervals. RPE ratings were asked after each time estimate. Three minutes of rest were given prior to beginning the 60% MVIC protocol. The order was not randomized as the 60% MVIC protocol could have induced some fatigue that could have affected the distraction (10% MVIC) protocol. As the distraction protocol would not induce fatigue, then it was determined that there would not be any adverse fatigue effects on the 60% MVIC by starting with a 10% MVIC protocol.

### Electromyography (EMG)

Since the SET and SBF theories suggest that time estimates are affected by the degree or extent of internal events (e.g., neuromuscular recruitment and rate coding), EMG was monitored to observe the relative change in neuromuscular activity with each condition. Before electrode placement, the skin was shaved, abraded, and cleansed with an isopropyl alcohol swab to reduce EMG recording impedance (24). EMG of the dominant quadriceps was monitored using self-adhesive 3.2 cm diameter Ag/AgCl bipolar electrodes (MeditraceTM 130 ECG, Syracuse, USA. The electrodes were placed parallel and edge-to-edge for an inter-electrode spacing of 20 mm over the rectus femoris mid-belly, midway between the anterior superior iliac spine and the patella's superior edge. A ground electrode was placed on the femoral lateral epicondyle. Following electrode placement, electrodes were taped to minimize movement and tested for inter-electrode impedance noise (<5 kOhms). All EMG signals were monitored (Biopac System Inc., DA 100: analog-digital converter MP150WSW; Holliston, Massachusetts) and recorded with a sampling rate of 2,000 Hz using AcqKnowledge III, Biopac System Inc software. EMG activity was filtered with a Blackman −61 dB band-pass filter between 10 and 500 Hz, amplified (bi-polar differential amplifier, input impedance = 2M*Ω*, common-mode rejection ratio > 110 dB min (50/60 Hz), gain × 1,000, noise > 5 µV), and analog-to-digitally converted (12 bit) and stored on a personal computer for further analysis. The integral of the 30-s rectified EMG signal was used for analysis.

### Tympanic temperature

Tympanic temperature was obtained and recorded by a tympanic thermometer (IRT6520CA ThermoScan, Braun, Germnay). The thermometer probe with a disposable plastic covering was inserted into the right ear canal to record the tympanic temperature. For each participant, the disposable plastic was changed. This procedure was performed on four occasions for each protocol: pre- and post-time familiarization protocol, pre-and post-test protocols.

### Heart rate

A heart rate monitor (T31 Heart Rate Sensor, Polar, USA) was used to obtain heart rate during pre-and post-time familiarization sessions and the time estimation protocol. The heart rate monitor was fixed using an elastic belt to be secured around the participant's third sternum.

### Rate of perceived exertion (RPE)

The RPE Borg Scale was used to rate the participant's physical activity intensity for each protocol on an increasing scale of 6–20. RPE was reviewed and asked during the control, maximal, submaximal, and distraction protocols to give insight if the participant was working at the correct intensity and to prevent participants from internal counting of time (seconds).

### Data analysis

Statistical analysis was completed using the SPSS software (Version 24.0, SPSS, Inc. Chicago, IL). First, normality (Kolmogorov-Smirnov) and homogeneity of variances (Levene) tests were conducted for all dependent variables. If the assumption of sphericity (Mauchly's test of sphericity) was violated, then the Greenhouse-Geisser correction was employed. Significance was established as *p* ≤ 0.05. A repeated-measures ANOVA was used to analyze the four conditions and time perception. Time estimation differences (deviation in time estimate from the chronological time: e.g., chronological time: 5-s, estimated time: 4.5-s, time estimation difference: −0.5-s) were analyzed for each time estimate (5-, 10-, 20-, 30-s) with a 4 × 2 × 2, 3-way ANOVAs, involving four conditions [control, maximal, 60% MVIC, and distraction (10% MVIC)] × 2 tests (pre-intervention and during the intervention) and a between factor of sex (female and male). Similarly, a 4 × 4 × 2, 3-way ANOVA was calculated for relative time differences with 4 conditions (control, maximal, 60% MVIC, and distraction (10% MVIC) × 4 times (5-, 10-, 20- and 30-s) with sex as a between factor. Normalized EMG signal was analyzed by conducting a 3 × 2 repeated measure ANOVA, activation of the dominant quadricep for the three exercising conditions [maximal, 60% MVIC, and distraction (10% MVIC)] x first and last 5 s of the approximate 30 s interval.

For heart rate and temperature, a three-way repeated-measured ANOVA was completed involving four intervention conditions [control, maximal, 60% MVIC, and distraction (10% MVIC)], and two times (pre- and post-intervention) and a between factor of sex. Paired t-tests with Bonferroni corrections were used to decompose significant interactions, and Bonferroni *post hoc* tests were used to determine main effect differences. Partial eta^2^ (*η*^2^) values were calculated with *η*^2^ = 0.01 indicating a small effect size, 0.06 indicating a medium effect size, and >0.14 indicating a large effect size for the main effects and interactions (27). Inter-session reliability of time estimates comparing pre-intervention values were assessed with Cronbach's alpha intraclass correlation coefficient (ICC) with correlation coefficients (CV). Data reported as mean ± SD.

## Results

All dependent variables were normally distributed according to the Kolmogorov-Smirnov test. All statistical details (F, *p*, and *n*^2^ values) are provided in Table 2.

**Table 2:** Overview of statistical main effects and interactions.

	Main effects for Time	Main effects for condition	Time x sex interaction	Condition x sex	Time x Condition
5-s time estimate	[F (1,17) = 18.644, *p* < 0.0001, *n*^2 ^= 0.523] Figure 2 Intervention < pre-intervention	[F (3,51) = 2.791, *p* = 0.00497, *n*^2 ^= 0.141] Figure 2 MVIC < Control	[F (1,17) = 6.476, *p* = 0.021, *n*^2 ^= 0.276] Female < Males	[F (1,17) = 24.89, *p* = 0.002, *n*^2 ^= 0.245] Female < Male @ 10% and 60% MVIC	NS
10-s time estimate	NS	NS	NS	NS	NS
20-s time estimate	[F (1,17) = 8.153, *p* = 0.011, *n*^2^ = 0.324] Figure 4 Intervention < pre-intervention	[F (3,51) = 3.149, *p* = 0.033, *n*^2^ = 0.156] Figure 4 MVIC < Control	[F (1,17) = 3.853, *p* = 0.06, *n*^2^ = 0.185] Female < Male	NS	[F (3,51) = 2.721, *p* = 0.05, *n*^2^ = 0.138] Table 3 MVIC < Control
30-s time estimate	[F (1,17) = 13.075, *p* = 0.002, *n*^2^ = 0.435] Figure 5 Intervention < pre-intervention	[F (3,51) = 5.038, *p* = 0.004, *n*^2^ = 0.229] Figure 5 MVIC < Control	[F (1,17) = 3.31, *p* = 0.08, *n*^2^ = 0.163] Female < Male	NS	[F (3,51) = 6.538, *p* = 0.001, *n*^2^ = 0.278] Table 3 MVIC and 60%MVIC <Control
Absolute time estimate differences	[F (3,54) = 5.29, *p* = 0.001, *n*^2^ = 0.258] 10-s < 5-,20-, 30-s	[F (3,54) = 3.519, *p* = 0.021, *n*^2^ = 0.164] Control < 60% and MVIC	NS	NS	[F (9,162) = 5.29, *p* < 0.0001, *n*^2^ = 0.227] Table 5
Relative (%) time estimate differences	[F(3,151) = 35.22; *p* < 0.0001; eta2: 0.674] Time underestimates at 5-s > 10-, 20-, and 30-s	NS	NS	NS	[F(9,153) = 3.15; *p* = 0.002; eta2: 0.156] Table 5
Heart Rate	NS	[F (3,51) = 25.59, *p* < 0.0001, *n*^2^ = 0.601] Control < 10%, 60% and MVIC	NS	[F (3,51) = 3.69, *p* = 0.017, *n*^2^ = 0.179] Control < 10%, 60% and MVIC	NS
Tympanic temperature	[F (1,17) = 32.19, *p* < 0.0001, *n*^2^ = 0.654] Pre-test < post-intervention	NS	NS	NS	NS

### Reliability

ICC calculations revealed generally good pre-test reliability for 5-s (ICC: 0.754, 4.75 ± 0.37-s, CV: 0.079), 10-s (ICC = 0.711, 10.17 ± 0.47-s, CV: 0.046), 20-s (ICC: 0.783, 20.58 ± 0.88-s, CV: 0.043) and 30-s estimates (ICC: 0.851, 30.83 ± 1.45-s, CV: 0.046).

### 5-seconds

**Figure 2:**
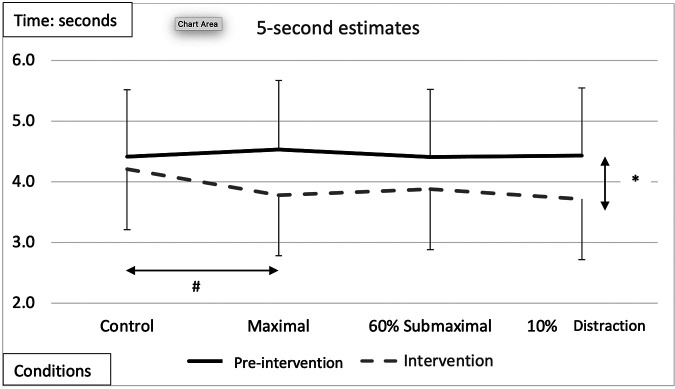
Subjective mean estimates for each condition for the five-second interval. Asterisks and double arrow represent a main effect for time (familiarization vs. intervention). Asterisks and double arrow illustrate main effect for conditions (familiarization and intervention values combined) with maximal significantly less than control condition.

A significant main effect of testing time for the pre-intervention and intervention conditions (Figure 2 and Tables 2, 3) showed greater underestimation of time for the intervention estimate at 5-s (−0.401 ± 0.93) compared to the pre-intervention. A significant main effect for conditions showed an underestimation of −0.445 ± 0.11-s (*p* = 0.006) between the control and maximal conditions (Figure 2 and Tables 2, 3). Additionally, a significant interaction effect for Time x Sex was highlighted with females demonstrating a greater underestimation (−0.372 ± 0.181) of 5 s during the interventions than males (Table 2). A condition x sex interaction revealed that women underestimated time compared to men with the 60% submaximal and 10% distraction conditions (Tables 2–4).

**Table 3:** Condition x time interaction time estimates mean (95% confidence intervals) (*n* = 19).

	Pre-5-s	Post-5-s	Pre-10-s	Post-10-s	Pre-20-s	Post-20-s	Pre-30-s	Post-30-s
Control	4.72 (4.56–4.86)	5.56 (4.33–4.79)	10.04 (9.84–10.24)	10.16 (9.78–10–53	20.34 (19.95–20.73	20.44 (19.74–21.14)	30.30 (29.74–30.87)	30.57 (29.38–31.75)
MVIC	4.80 (4.63–4.98)	**4.43**^b^ **(4.06–4.80)**	10.06 (9.79–10.32)	9.87 (9.03–10.71)	20.28 (19.94–20.62)	**18.59**^b^ (**17.33–19.85)**	30.32 (29.71–30.94)	**27.42**^b^ (**25.45–29.37)**
60% MVIC	4.74 (4.52–4.96)	4.27 (3.85–4.68)	10.31 (10.07–10.53)	10.01 (9.38–10.64)	20.84 (20.33–21.35)	18.99 (17.88–20.11)	31.35 (30.49–32.21)	**27.38**^b^ (**25.85–28.91)**
10% MVIC (distraction)	4.76 (4.56–4.96)	4.11 (3.76–4.45)	10.28 (10.03–10.53)	10.23 (9.19–11.27)	20.85 (20.33–21.36)	20.09 (18.45–21.73)	31.33 (30.44–32.22)	29.56 (27.65–31.47)
Main effects for Time	4.75 (4.56–4.98)	**4.59**^a^ (**3.76–4.80)**	10.17 (9.79–10.53)	10.06 (9.03–11.27)	20.57 (19.94–21.36)	**19.52**^a^ (**17.33–21.73)**	30.82 (29.71–30.94)	28.73 (25.45–31.75)

Bolded figures highlight significant differences.

^a^ Indicate a significant main effect for time for the specific time intervals (i.e., 5- and 20-s).

^b^ Indicate a significant difference from the control condition for the testing time in the specified column [i.e., MVIC (maximal voluntary isometric contraction) post-5-, 20-, 30-s, 60% MVIC post-30-s].

**Table 4:** Sex differences: average estimated 5-s for males, females, and total for each condition during familiarization and intervention (mean ± SD).

	Control		Maximal		60% MVIC Submaximal		10% MVIC Distraction	
	Practice	P-P	Practice	P-P	Practice	P-P	Practice	P-P
Males	4.67 ± .11	4.68 ± .16	4.67 ± .12	4.49 ± .26	4.75 ± .16	4.61* ± .27	4.73 ± .14	4.39* ± .23
Females	4.76 ± .10	4.46 ± .15	4.93 ± .11	4.38 ± .25	4.74 ± .15	3.97* ± .26	4.79 ± .14	3.86* ± .22
Average	4.72 ± .10	4.57 ± .16	4.80 ± .12	4.43 ± .26	4.75 ± .16	4.29 ± .27	4.76 ± .14	4.13 ± .23

P-P, post-protocol. Bolded with asterisk values indicate significant differences in time perception between males and females.

### 10-seconds

**Figure 3:**
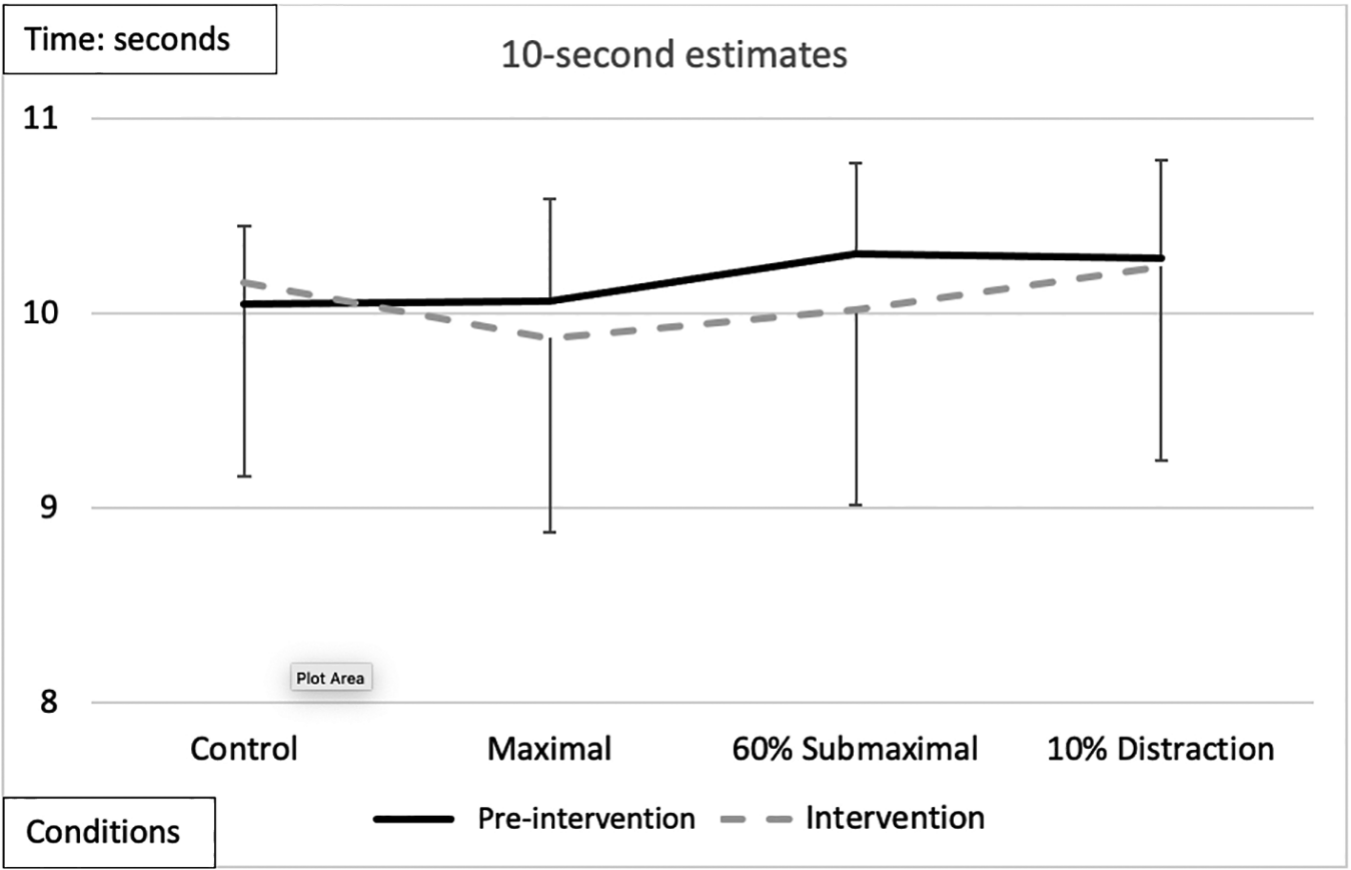
Subjective mean estimates for each condition for the 10-s interval. There were no significant main effects or interactions.

There were no significant main effects or interactions [F(3,51) = 0.164, *p* = 0.920, *n*^2 ^= 0.010] between conditions [F(3,51) = 0.254, *p* = 0.858, *n*^2 ^= 0.015], time [F(1,17) = 0.207, *p* = 0.526, *n*^2 ^= 0.184], or sex [F(1,17) = 0.828, *p* = 0.376, *n*^2 ^= 0.046] (Figure 3).

### 20-seconds

**Figure 4:**
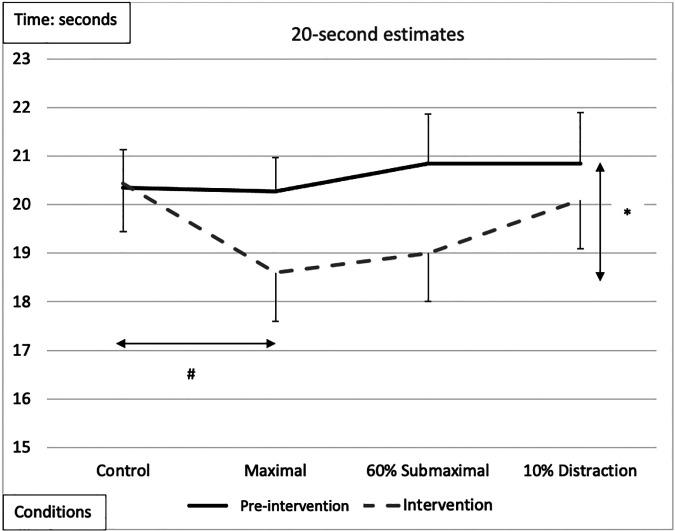
Subjective mean estimates for each condition for the 20-s interval. Asterisks and double arrow represent a main effect for time (familiarization vs. intervention). Asterisks and double arrow illustrate main effect for conditions (familiarization and intervention values combined) with maximal significantly less than control condition.

There was a significant main effect for testing time with an overall underestimation of time by −0.48 *± *0.26-s during the interventions compared to the pre-intervention (Figure 4 and Tables 2, 3). A significant main effect for conditions demonstrated that during the maximal condition, time was underestimated to a greater degree than the control condition (*−0.862 ± 0.24, p = 0.016)* (Figure 4 and Tables 2, 3). A significant Time x Condition interaction indicated that the maximal condition underestimated the 20 s time interval by an average of −1.813 ***± ***0.51-s (*p* = 0.013) compared to the control condition during the intervention (Figure 4). A large effect size magnitude, but non-significant Time x Sex interaction suggested that females underestimated the 20-s time interval to a greater extent than males during the intervention (females: −1.703* ±* 0.32-s vs. males: −0.315* ± *0.28-s) (Table 2).

### 30-seconds

**Figure 5:**
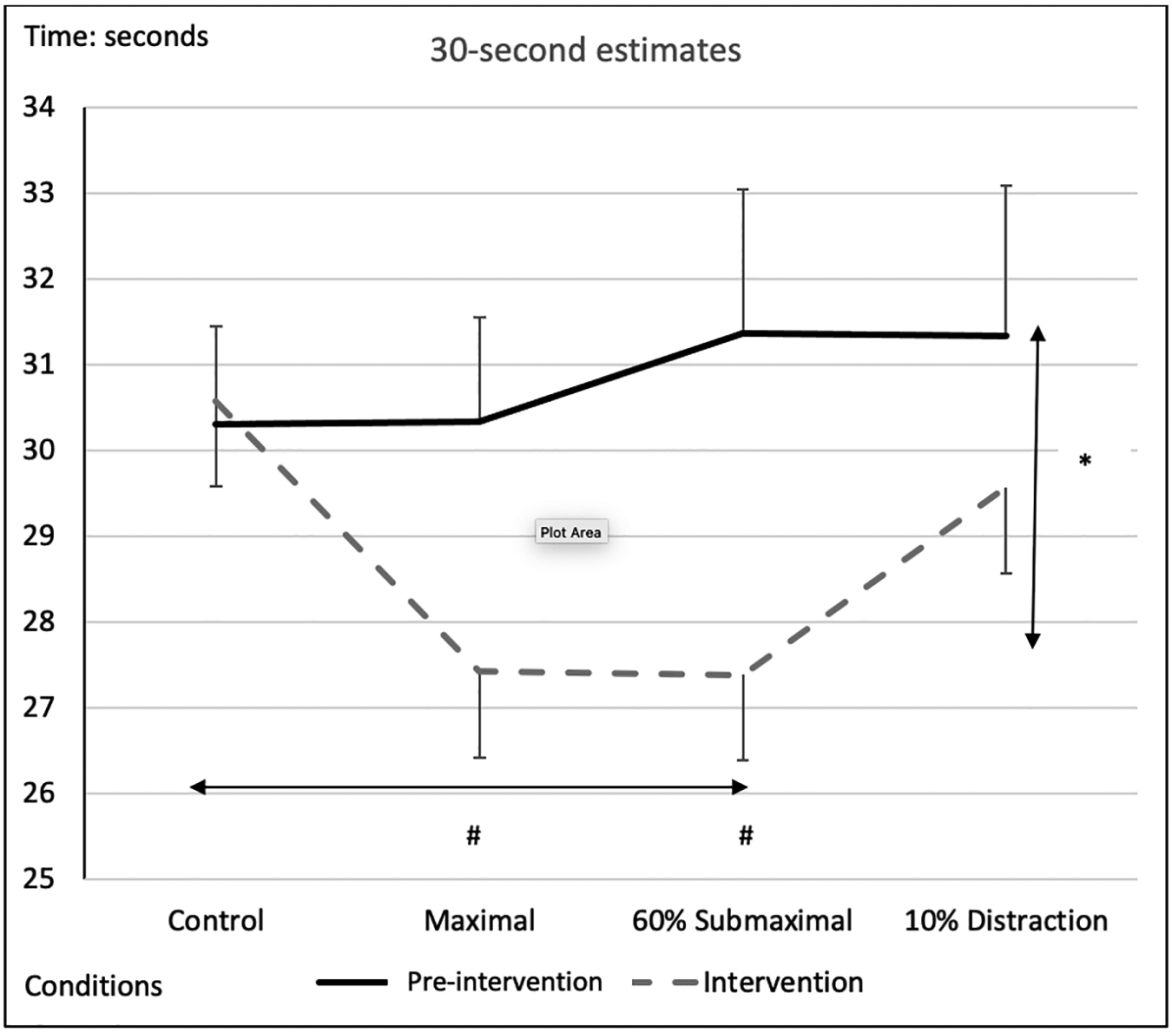
Subjective mean estimates for each condition for the five-second interval. Asterisks and double arrow represent a main effect for time (familiarization vs. intervention). Asterisks and double arrow illustrate main effect for conditions (familiarization and intervention values combined) with maximal and 60% submaximal significantly less than control condition.

There was a significant main effect for testing time between pre-intervention and intervention protocols, with an overall underestimation of time by −2.04 *± *0.54-s during the interventions compared to the pre-intervention test (Figure 5 and Tables 2, 3). A significant main effect for conditions demonstrated that during the maximal condition, time was underestimated to a greater degree than the control condition (−1.528 ± 0.35-s, *p* = 0.003) (Figure 5 and Tables 2, 3). A significant Time x Condition interaction exhibited that the maximal and 60% submaximal conditions underestimated time during the intervention by −2.83 *±* 0.56-s *(p = 0.01)* and −3.88 *±* 0.68-s *(p = 0.028)* respectively compared to an overestimation during the control condition (+0.242 *±* 0.25-s) (Table 2). Furthermore, there was another large effect size magnitude, but non-significant Time x Sex interaction indicating that females showed a greater underestimation of time (males: −1.01 *±* 0.48-s vs. females: −3.07 *±* 0.58-s) (Table 2).

### Absolute time estimate differences

A significant main effect for conditions (all time estimates combined) revealed a greater underestimation of time during the 60% MVIC (−1.08-s *±* 1.61; *p* = 0.033) and MVIC (−1.17-s *±* 2.16; *p* = 0.026) conditions compared to control (0.18-s *±* 1.13) (Table 2). Furthermore, a significant main effect for time indicated that difference in time estimation for 10-s (0.073-s *±* 1.07) was less than 5- (−0.65-s *±* 0.57; *p* = 0.001), 20- (−0.465-s *±* 1.66; *p* = 0.06) and 30-s (−1.265-s *±* 2.41; *p* = 0.021). Thirty seconds estimate deviations were greater then 20-s estimates (*p* = 0.046). A significant condition x time interaction was apparent with time estimate deviations from the chronological time for the Control and MVIC 5-s estimates exceeding the 10%MVIC. The control condition 20- and 30-s perceptions overestimated time compared to MVIC and 60% MVIC. The 30-s estimate was significantly lower (greater underestimation) than the 10% MVIC condition whereas the 60% MVIC 30-s estimate also had a greater underestimation compared to the 10% MVIC condition (Table 5).

**Table 5:** Condition x time interactions.

Absolute Time Difference (s)	5-seconds	10-seconds	20-seconds	30-seconds
Control	−0.43 ± 0.46 *p* = 0.0009* *p* = 0.08^#^	0.16 ± 0.75	0.44 ± 1.41 *p* = 0.002* p = 0.03 #	0.57 ± 2.39 *p* = 0.001* p = 0.001^#^
MVIC	−0.56 ± 0.74 *p* = 0.08 *α*	−0.12 ± 1.69	−1.40 ± 2.54*	−0.72 ± 0.83* p = 0.05 α
60% MVIC	−0.72 ± 0.83^#^	0.016 ± 1.26	−1.00 ± 2.25^#^	−2.61 ± 3.09^#^ *p* = 0.02 *β*
10% MVIC	−0.88 ± 0.69* α	0.23 ± 2.1	0.09 ± 3.31	−0.43 ± 3.85 α β
Relative Time Difference (%)				
Control	−8.7% ± 9.3 *p* = 0.08 *χ* *p* = 0.0009 *δ*	1.6% ± 7.5	2.2% ± 7.1 *p* = 0.002 χ	1.9% ± 7.9 *p* = 0.001 χ *p* = 0.001 δ
MVIC	−11.3% ± 14.9	−1.2% ± 16.9	−7.0% ± 12.7 χ	−8.6% ± 13.2 χ *p* = 0.05 *ϕ*
60% MVIC	−14.5% ± 16.7 χ	0.16% ± 12.6	5.0% ± 11.2	−8.7% ± 10.3 δ *p* = 0.018 *γ*
10% MVIC	−17.7% ± 13.9 δ	2.3% ± 20.9	0.48% ± 16.5	−1.4% ± 12.8 ϕ γ

The specific symbols (i.e., *, # and Greek symbols) highlight significant differences between the two conditions with the same symbol within the column.

### Relative time estimate differences

When examining the relative (%) time estimate differences (absolute time estimate difference / the chronological time), there was a significant main effect for time (Table 2) with greater relative underestimations of time at 5-s (−12.9% ± 11.3) vs. 10-s (*p* < 0.0001; 0.8% ± 10.8), 20-s (*p* < 0.0001; −2.2% ± 8.2) and 30-s (*p* = 0.001; −4.1% ± 7.8). There was also a significant (*p* = 0.045) relative underestimation at 30-s compared to an overestimation at 10-s. Significant condition x time interactions (Table 2) revealed less time underestimations with Control at 5-s vs. 10% and 60% MVIC. At 20-s, the MVIC conditions presented a significant relative time underestimation compared to an overestimation with the control condition. Similarly, the overestimation of Control at 30-s significantly varied from the underestimations with MVIC, 60% MVIC and 10% MVIC conditions (Table 5).

### Heart rate

A significant main effect for condition showed that the control condition exhibited lower heart rate (75.3 *± *11.6) than the maximal (92.5 *±* 13.9), 60% submaximal (92.2 *±* 14.4) or distraction (90.5 *±* 14.7) conditions (Table 2). Post hoc pairwise comparisons also revealed that heart rate was higher in the submaximal condition vs. the distraction protocol. A condition x sex interaction similarly showed that for both men and women, the control condition experienced lower heart rate than for the experimental conditions (Table 2).

### Tympanic temperature

There was a main effect for time with post-tests (36.67 ± 0.25°C) exceeding pre-tests (36.45 ± 0.33°C) temperatures (Table 2).

### Relative (normalized) EMG

There was a significant effect for dominant leg EMG x condition [F (2,36) = 45.794, *p* = 0.001, *n*^2^ = .718], which showed a lower activation of the dominant quadriceps during the 10% distraction protocol than the maximal and submaximal conditions. A pairwise comparison test reported that the mean difference was 2.41 mV.s (*p* < 0.0001) and 1.71 mV.s (*p* < 0.0001) between the maximal and submaximal vs. distraction conditions respectively.

## Discussion

To our present knowledge, this is the first study to investigate the temporal perception of time intervals in humans while performing varying intensity isometric contractions and possible sex differences. The primary findings include that as chronological time increases, time perception is underestimated during submaximal and maximal exercise. The integrated EMG signal was reported to be greater during the maximal and submaximal contractions than the distraction condition, possibly indicating a greater physiological arousal. The maximal (at 5-, 20-, and 30-s) and submaximal (30-s) isometric contractions impaired time perception more than the control and distraction conditions. Underestimation of absolute time was most evident during the maximal condition at the 30-s interval. Although, with all conditions combined, the relative (%) underestimation of time was greatest at the 5-s estimate. The distortion of time due to exercise has been a similar finding in past research (10, 20, 28). Furthermore, there was a significant underestimation of time by females at 5-s with similar, but near non-significant underestimations at 20-s (*p* = 0.06) and 30-s (*p* = 0.08).

The variation of time estimates was most pronounced at the 30-s interval while performing a dual-task. Gazes et al. (29) found a reduction in cognitive and motor tasks when performed together. Similarly, Polti and colleagues (30) found a significant underestimation of time for 30-s to 90-s intervals when participants performed a dual-task. The present study had participants encounter a cognitive-motor dual-task that revealed an influence on time perception. The cognitive-motor interference has shown to distort time intervals as the two tasks are simultaneously being performed, where one or both performances are impaired (31). In comparison, Brown (32) concluded that when performing a dual-task, the judgment of the time interval varied to a greater degree with prolonged chronological time. The attentional gate model can explain the misjudgment of time, a model relating attentional allocation and estimating time from the SET (33). When the task's priority is temporal processing, the attentional gate allows pulses to enter cognition for an accurate estimation. Conversely, when a non-temporal processing task (such as exercise) is devoted, the attentional gate is narrowed, allowing fewer pulses to enter and impairing time perception.

Absolute time variation tended to be more pronounced with the maximal and submaximal intensity contraction conditions, particularly at 30-s, where estimates were shorter (underestimated) than with chronological time. Normalized EMG signals revealed greater mean amplitudes during the maximal and submaximal contraction resulting in increased corticospinal excitability and motor unit activation (14). During muscle contractions, research has shown that neural output increases (motor unit recruitment, rate coding, synchronization, muscle action potential conduction velocity, and increased hormones such as dopamine) to reinforce the muscle for proper performance (34, 35). In turn, this increase in neurophysiological events within the given time frame will increase the proposed mechanisms of time perception SET or SBF to underestimate time intervals.

Edwards and McCormick (28) reported similar findings, as participants that exercised at a higher intensity (RPE 20) underestimated time to a greater degree. Findings from this study state that at the 5- and 10-s interval mark, time perception did not significantly differ between exercising conditions. However, at 20- and 30-s, there was a significant difference between the exercising conditions, where the maximal protocol (*p* = 0.003) caused an underestimation to a greater extent. Likewise, Benko and Cimrová (20) instructed participants to estimate 10-, 15-, and 20-s when performing a hand-grip test for nine pseudo-randomized sequences, with and without an inflatable cuff to cause arm occlusion. The researchers found that overstimulation of visceroreceptors from isometric contractions can distort temporal intervals. Therefore, time underestimation was distinct in response to maximal efforts and longer time intervals. At higher intensities of exercise, catecholamines release into the blood where there is greater sensory awareness of physical discomfort. As a result, neural networks will have greater activity, which may result in a time distortion, as explained by the SET. The SET would depict that the increase in arousal and change in visceroreceptors potentially increases the internal clock's pace and further skews perception of time (3).

The results from the control condition showed that a dual-task of estimating time and reporting RPE have less time estimation variation compared to the other three conditions. A non-temporal task, such as exercise, can result in hyperarousal, where the body experiences greater sensory awareness of physical discomfort. As previously mentioned, a possible explanation is that an MVIC such as a knee extension is an intense contraction that will increase motor unit recruitment, rate coding, intermuscular coordination, and other factors that result in greater force development (36). During the exercise conditions, participants focused on working at the correct intensity while concurrently estimating time intervals. The body experiences higher arousal from the intense isometric contraction. The higher arousal would result in a greater amount of brain processing, and attention to the alternate task is distorted. The control condition was not as physically taxing to the body compared to the other three exercising conditions allowing more focus on the cognitive task. Though deviations were reported in the familiarization and intervention estimations, this may be due to arousal experienced as researchers asked participants to perform a task. Wearden (37) reported similar findings, in that the distortion of time occurs with increased arousal. Furthermore, they concluded that this time distortion may be due to the SET as the rapid timing interval pulses are compared to the initially encoded control condition.

Increases in body temperature have been suggested to distort temporal perception (38, 39), with even minor circadian increases (in the afternoon) causing an overestimation of time (40) or water immersion at 38°C resulting in underestimated time intervals (41). Tamm et al. (42) reported temporal compression as the core temperature increased when participants ran in a warm, humid environment. Although in the present study, there was a significant increase in body temperature over time (pre- to post-tests), there were no significant differences between contraction conditions. For tympanic temperature to be a moderating factor, it would be expected that higher temperatures would have corresponded with the underestimation of time with maximal (time underestimation at 5-, 20- and 30-s) and submaximal contractions (time underestimation at 30-s), but there were no significant differences. Similarly, there was no significant differences in HR between maximal and 60% submaximal contractions even though time estimates were significantly underestimated with maximal contractions compared to submaximal at 5-s and 20-s. In summary, HR measures presented some greater sensitivity than body temperature as HR was lower with control vs. MVIC and 60% MVIC as well as lower with the distraction condition (10% MVIC) than the 60% MVIC, whereas body temperature analysis demonstrated an overall time effect but no differentiation between conditions. Thus, neither measure was a highly sensitive indication of changes in arousal (e.g., HR: no significant difference between MIC and 60% MVIC) and any changes may not have been substantial enough to alter time estimates.

When not considering the different contraction intensity conditions, the finding indicated greater relative (% differences) time underestimations overall (all conditions combined) for the 5-s period. These results might suggest that the absolute deviations in time estimates with longer durations might be associated with the accumulation of time estimate differences over the four time periods. For example, if the estimate at each time period was incorrect by 0.5-s then the 5-s estimate would show a 0.5 s deviation (10%), whereas the sum of these additional 5-s deviations for 30-s estimate (0.5-s deviations each for 5-, 10-, 20- and 30-s) would be offset by 2-s (6.6%) resulting in a relatively lower time estimate deviation. Furthermore, the greater overall relative time underestimations at 5-s might again be related to arousal. Similar to a start of a race, the start of the time interval test within an unfamiliar lab environment in the presence of two researchers may have induced some anticipatory excitation, increasing sympathetic stimulation and catecholamines, which could possibly have adversely affected time perception. As the time interval test continued, the sympathetic excitation may have diminished to some extent. Alternatively, perhaps our participants and people in general are more accustomed or trained to estimate longer durations such as 10- to 30-s. When examining the relative differences, there were still greater time estimate deviations with the contraction conditions vs. the control (Table 5) but no significant differences between times with the exception of the 10-s estimates.

It was intriguing that subjects underestimated time more at 5-, 20-, and 30-s compared to 10-s. The rationale may be speculated to be related to common lifelong experiences and learning. Countdowns from 10-s are common with for example, televised rocket take-offs to space, the countdown to the New Year, the final duration of an exercise and the end of many time restricted sports (e.g., basketball, tennis, ice hockey, football). As the subjects in this study were recreationally active, they may also be accustomed to 10-s intervals from sports and exercise, where it is common for a trainer to push athletes by saying, “only 10-s remaining”. It is possible that this additional lifelong exposure of 10-s time intervals led subjects to estimate the 10-s time point most accurately.

There was a significant sex difference reported with underestimated temporal estimations at 5-s for females whereas there were near but non-significant underestimations at the 20- (*p* = 0.06) and 30-s (*p* = 0.08) time intervals. Previous literature has shown differences in females and males while others have not (22, 43, 44). Similar to the present study, Hanson and Buckworth (22), found that females tend to underestimate time more than males during a self-paced run. Although, females ran at a higher self-pace resulting in the two groups exercising at different intensities. As well, our study analyzed intervals of 5-, 10-, 20-, and 30-s before and during an isometric knee extension, while Hanson and Buckworth (22) performed assessments before, during, and after a running protocol.

Whereas females are reported to exhibit greater fatigue resistance with prolonged, aerobic type activities due to a greater emphasis on fat metabolism, lower type II muscle fibre composition (45, 46), lesser muscle deoxygenation (47) and higher muscle perfusion (48), men have exhibited greater fatigue resistance with high intensity exercise (49). In addition, there are reported sex-related differences in dopamine (50) and GABA neurotransmitters (51), which could influence the SBF theory control of time perception. Women are also reported to be more affected by attentional and arousal stress (52) as would have been experienced with the expectations to maintain the prescribed voluntary contractions and estimate time concurrently. Hence, greater fatigue effects with the isometric contractions, in addition to the sex differences in neurotransmitters and greater sensitivity to attentional and arousal stress could have contributed to greater time estimate deviations by women in this study. Further investigation into sex differences in time perception during exercise is needed to add to the small pool of research.

This study had limitations. Since participants were trained young females and males, the results may not be representative of untrained or older individuals. Likewise, as this study was conducted in a laboratory where participants underwent isometric knee extension contractions, it is unknown whether these findings would translate into real-life tasks or competitions. Although, the 19 participants exceeded the participant number required to achieve sufficient statistical power as calculated with an “a priori” statistical power analysis (G*Power), a great number of participants would always provide stronger results.

## Conclusions

This study found that time perception was impaired when performing isometric knee extension contractions at maximal (at 5-, 20- and 30-s) and 60% submaximal (30-s) intensities. The deficits were most evident during the maximal contraction intensity condition at 30-s. These results add to the growing body of literature on time perception and exercise. With many athletes competing at a high intensity, this may suggest that their perception of time is negatively skewed (typically underestimated). Likewise, exercise programs may be more enjoyable with high-intensity exercises for a 30-s interval, as individuals may believe that time is “flying by” (accelerated). Future research should aim to investigate time perception on types of contractions, sex differences, and compare athletic to non-athletic populations. This study is a great foundation for understanding time perception in humans when enduring a short bout of isometric exercise.

## Data Availability

The original contributions presented in the study are included in the article/Supplementary Material, further inquiries can be directed to the corresponding author.
